# Mitigating Motor Neuronal Loss in *C. elegans* Model of ALS8

**DOI:** 10.1038/s41598-017-11798-6

**Published:** 2017-09-14

**Authors:** Wendy Zhang, Antonio Colavita, Johnny K. Ngsee

**Affiliations:** Neuroscience, Ottawa Hospital Research Institute, Cellular and Molecular Medicine, University of Ottawa, 451 Smyth Road, Ottawa, Ontario, Canada K1H 8M5

## Abstract

ALS8 is a late-onset familial autosomal dominant form of Amyotrophic Lateral Sclerosis (ALS) caused by a point mutation (P56S) in the VAPB gene (VAMP associated protein isoform B). Here, we generated two *C*. *elegans* models of the disease: a transgenic model where human VAPB wild-type (WT) or P56S mutant was expressed in a subset of motor neurons, and a second model that targeted inducible knockdown of the worm’s orthologue, *vpr-*1. Overexpression of human VAPB in DA neurons caused a backward locomotion defect, axonal misguidance, and premature neuronal death. Knockdown of *vpr-*1 recapitulated the reduction in VAPB expression associated with sporadic cases of human ALS. It also caused backward locomotion defects as well as an uncoordinated phenotype, and age-dependent, progressive motor neuronal death. Furthermore, inhibiting phosphatidylinositol-4 (PtdIns 4)-kinase activity with PIK-93 reduced the incidence of DA motor neuron loss and improved backward locomotion. This supports the loss of VAPB function in ALS8 pathogenesis and suggests that reducing intracellular PtdIns4P might be an effective therapeutic strategy in delaying progressive loss of motor neurons.

## Introduction

Amyotrophic Lateral Sclerosis (ALS) is a late-onset, progressive neurodegenerative disease that results in rapid loss of upper and lower motor neurons. A point mutation in the vesicle-associated membrane protein (VAMP)/synaptobrevin-associated membrane protein B gene (*VAPB)* causes late-onset familial ALS8^1^. This mutation was first identified in a large Brazilian family, but the same mutation is found in European^2^ and Asian^3^ populations. The C → T transition results in substitution of the conserved proline by a serine (P56S). VAPB is a type II integral membrane protein localized to the endoplasmic reticulum (ER) and ER-Golgi intermediate compartment (ERGIC). The P56S mutation causes the protein to be highly prone to aggregation^4^. Since VAPB functions as homodimers, dimerization of wild-type VAPB with the mutant aggregate leads to its loss of function. Thus, mutant VAPB acts in a dominant negative fashion and produces a phenotype similar to the knockdown of VAPB^5^.

The VAPs are phylogenetically conserved from yeast to mammals. *C*. *elegans* has a single orthologue of the VAPs, *vpr-1*. Its highly conserved N-terminal Major Sperm Protein (MSP) domain consists of a seven-stranded β sheet sandwich and shares 81% similarity to human VAPB^6^. While the worm’s *msp* genes are expressed only in late primary spermatocytes, *vpr-1* is ubiquitously expressed and knockout of *vpr-1* is embryonic lethal^7–10^. Of the 302 neurons in *C*. *elegans*, 113 neurons are motor neurons that control locomotor behaviour such as crawling, thrashing or swimming, and motility of the reproductive and alimentary systems^11, 12^. “Dorsal A” (DA) and “dorsal B” (DB) cholinergic motor neurons are classes of motor neurons that are easily visible and often used in worm neuronal studies. These are ventral cord motor neurons that innervate dorsal muscles by sending commissures to the dorsal side^12^. The nine DA motor neurons, with commissures directed towards the anterior end, are responsible for backward locomotion, while the seven DB neurons with commissures directed towards the posterior end are responsible for forward locomotion^11, 12^. Although *C*. *elegans* move in a forward sinusoidal motion, its movement is interrupted with occasional turns and reversals. Immediate reversals in locomotion can also be induced upon gentle touch stimulus at the anterior body^13^.

To characterize the role of VAPB in motor neurons, we generated two *C*. *elegans* models: one expressing human VAPB-WT or P56S, and another with the knockdown of *C*. *elegans vpr-1*. In both models, disruption of VAPB function was targeted to DA motor neurons. Overexpression of human VAPB in DA neurons caused backward locomotion defects and premature neuronal death. Moreover, overexpression of WT and P56S unexpectedly caused axonal guidance defects. Knockdown of *vpr-1* in *C*. *elegans* also caused backward locomotion defects as well as age-dependent motor neuronal death. The more posterior DA6 and DA7 neurons are the most vulnerable in both models. Treatment with the phosphatidylinositol-4 (PtdIns4)-kinase inhibitor, PIK-93, reduced the incidence of DA neuronal loss and partially rescued backward-directed locomotor defects, suggesting restoration of phosphatidylinositol-4-phosphate (PtdIns4P) homeostasis may bypass VPR-1 function.

## Results

### Transgenic worms expressing human VAPB in DA motor neurons display an age-dependent backward locomotion defect

To model motor neuron degeneration in human ALS8, we first generated transgenic *C*. *elegans* overexpressing human VAPB-WT and VAPB-P56S under the *unc-4* promoter in DA and VA motor neurons, which control backward locomotion. Upon inducing a gentle touch stimulus to the head, the worm changes from forward to backward locomotion through a change in the direction of propagation of the sinusoidal body bends called “reversal behaviour”. Reversal behaviour assays were conducted on the transgenic worms by counting the number of body bends backward and the length of time it took between applying the stimulus and the subsequent return to forward locomotion.

**Figure 1:**
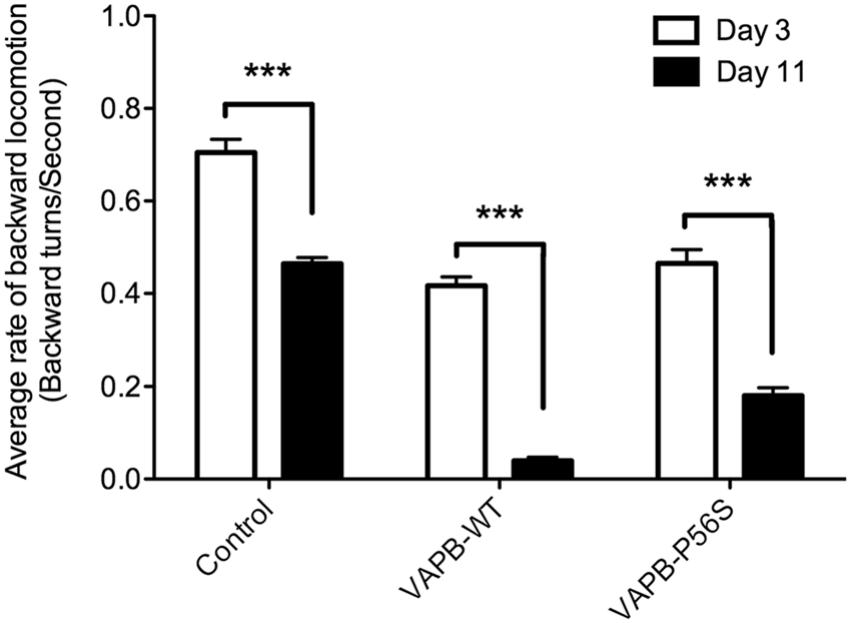
VAPB-WT and VAPB-P56S transgenic *C. elegans* strains exhibit a decreased rate of backward locomotion that worsens with age. Rate of backward locomotion of worms ages Day 3 and 11 of VAPB transgenic strains. Two-way ANOVA analysis showed that there is a significant interaction between strains and age with the rate of backward locomotion. Further Bonferroni post-test analysis determined the significant difference between Day 3 and 11 within the same strain. ***Represents p < 0.001. Comparison of VAPB-WT and P56S on Day 11 is also significant. Average range n = 100–150, repeated 3 times. Error bars represent standard error of the mean.

Multiple transgenic strains carrying extrachromosomal arrays were examined. Although all strains were morphologically normal, both adult Day 3 VAPB-WT and VAPB-P56S worms exhibited fewer body bends over a longer duration of time (0.42 ± 0.02 turns/second and 0.47 ± 0.03 turns/second, respectively), which resulted in a significant decrease in the rate of backward locomotion compared to the control (0.71 ± 0.03 turns/second, p < 0.001) (Fig. 1). The rate of backward locomotion decreased with age in all worms at adult Day 11, but the decline was more pronounced in worms overexpressing VAPB-WT or VAPB-P56S, and both were significantly lower than control. The defect with VAPB-WT (0.04 ± 0.01 backward turns/second) was significantly worse than VAPB-P56S worms (0.18 ± 0.02 backward turns/second; p < 0.001) at Day 11. Thus, overexpression of VAPB-WT or VAPB-P56S in DA neurons caused a locomotor defect that worsened with age.

### VAPB causes axonal misguidance of DA motor neurons

**Figure 2:**
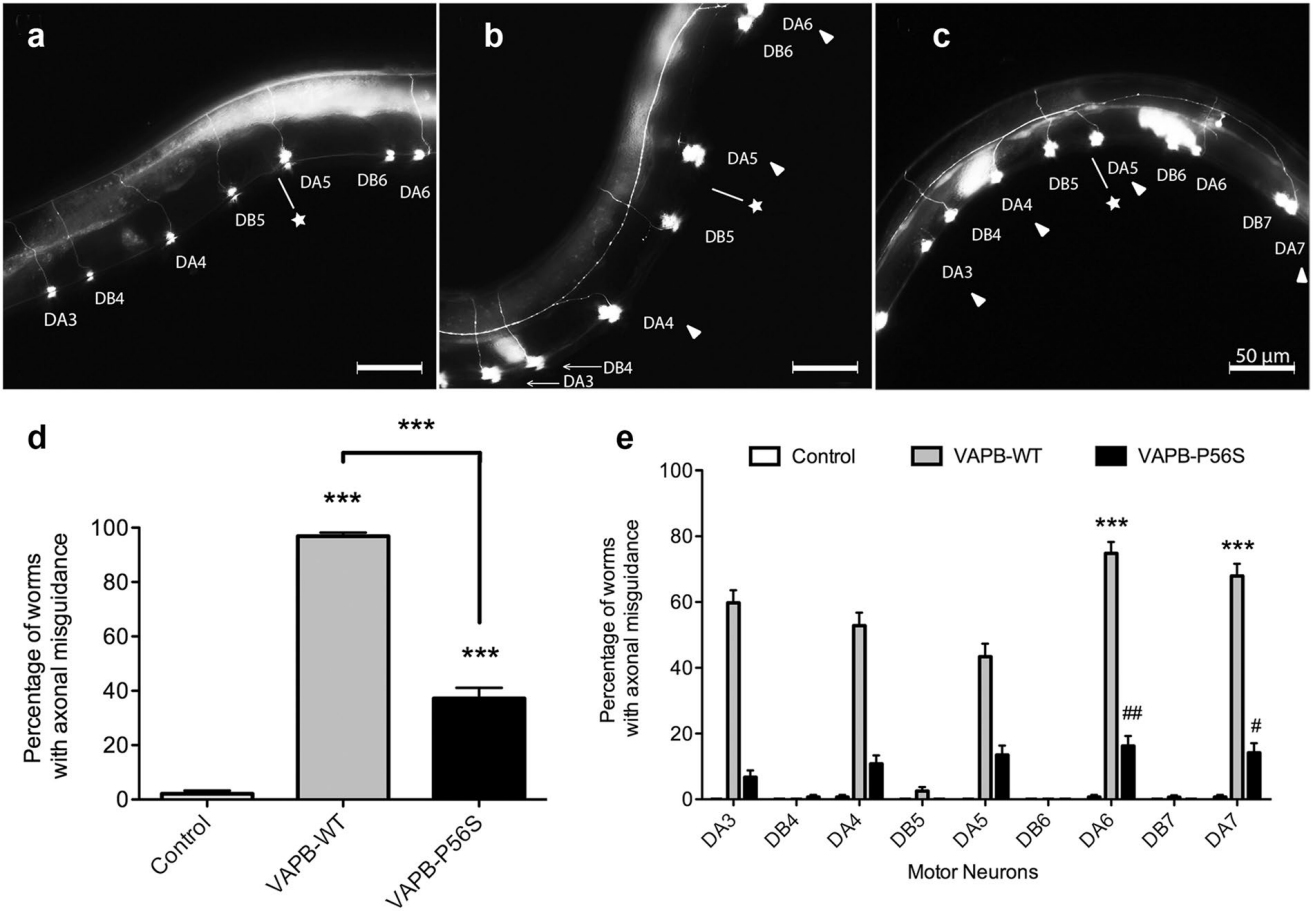
VAPB overexpression causes axonal misguidance in DA motor neurons. Representative images of DA and DB neurons of (**a**) control and worms expressing (**b**) FLAG-tagged human VAPB-WT and (**c**) VAPB-P56S at Day 3 of adulthood. Arrowheads represent misguided axons and position of the vulva is represented by a star symbol. (**d**) Average frequency of transgenic VAPB worms exhibiting axonal misguidance in DA neurons at Day 3. ***Represents p < 0.001, as determined by one-way ANOVA, followed by Bonferroni post-tests. (**e**) Incidence of axonal misguidance in DA and DB neurons at Day 3. Two-way ANOVA analysis showed that there is a significant interaction between specific DA neurons and strains with the percentage of axonal misguidance. Further Bonferroni post-test analysis determined the significant difference between axonal misguidance of specific neurons within the same strain. ***Represents p < 0.001 across DA neurons in VAPB-WT; ## represents p < 0.01 and # represents p < 0.05 across DA neurons in VAPB-P56S. Average range n = 100–150, repeated 3 times. Error bars represent standard error of the mean.

We next determined whether the backward locomotion defect is associated with degeneration of DA neurons. To visualize the affected motor neurons, the transgenic strains were crossed to the reporter strain, *unc-129p::GFP*, which expresses GFP under the *unc-129* promoter in the DA and DB motor neurons^14^. The axons of DA and DB neurons are parallel to each other and project from the cell body on the ventral cord to the dorsal midline in control worms (Fig. 2a). In contrast, axonal misguidance was observed at high frequency in both VAPB-WT (Fig. 2b) and VAPB-P56S (Fig. 2c). Axons projecting from the cell bodies of certain DA motor neurons were mistargeted longitudinally along the lateral body wall instead of projecting directly to the dorsal midline. VAPB-WT and VAPB-P56S had significantly higher incidences of worms with one or more axons that were misguided at 96.9% ± 1.4% and 37.2% ± 4.0%, respectively, compared to control (2.1% ± 1.2%, p < 0.001). Interestingly, VAPB-WT worms had the highest frequency of misguidance (Fig. 2d). Axonal misguidance was also evident at larval L4 stage of the VAPB-WT transgenic worms (Supplemental Fig. 1), indicating that this is a developmental defect.

The *unc-4* promoter drives expression of VAPB-WT and VAPB-P56S solely in class A neurons. Consistently, axonal misguidance was observed mainly in DA neurons with little to no misguidance in DB neurons (Fig. 2e). Various DA neurons exhibited axonal misguidance, but the most susceptible neurons are the more posteriorly located DA6 and DA7 neurons, affecting 74.8% ± 3.4% and 67.9% ± 3.7% of VAPB-WT and VAPB-P56S worms, respectively (Fig. 2e). This suggests that both VAPB-WT and VAPB-P56S overexpression lead to neuronal dysfunction in DA motor neurons with the posterior DA6 and DA7 motor neurons most susceptible to axonal misguidance in the VAPB-WT strain. This unexpected defect suggests that cellular levels of VAPB may play a role in axonal guidance in the worm.

### VAPB-WT and VAPB-P56S cause progressive degeneration of DA motor neurons

**Figure 3:**
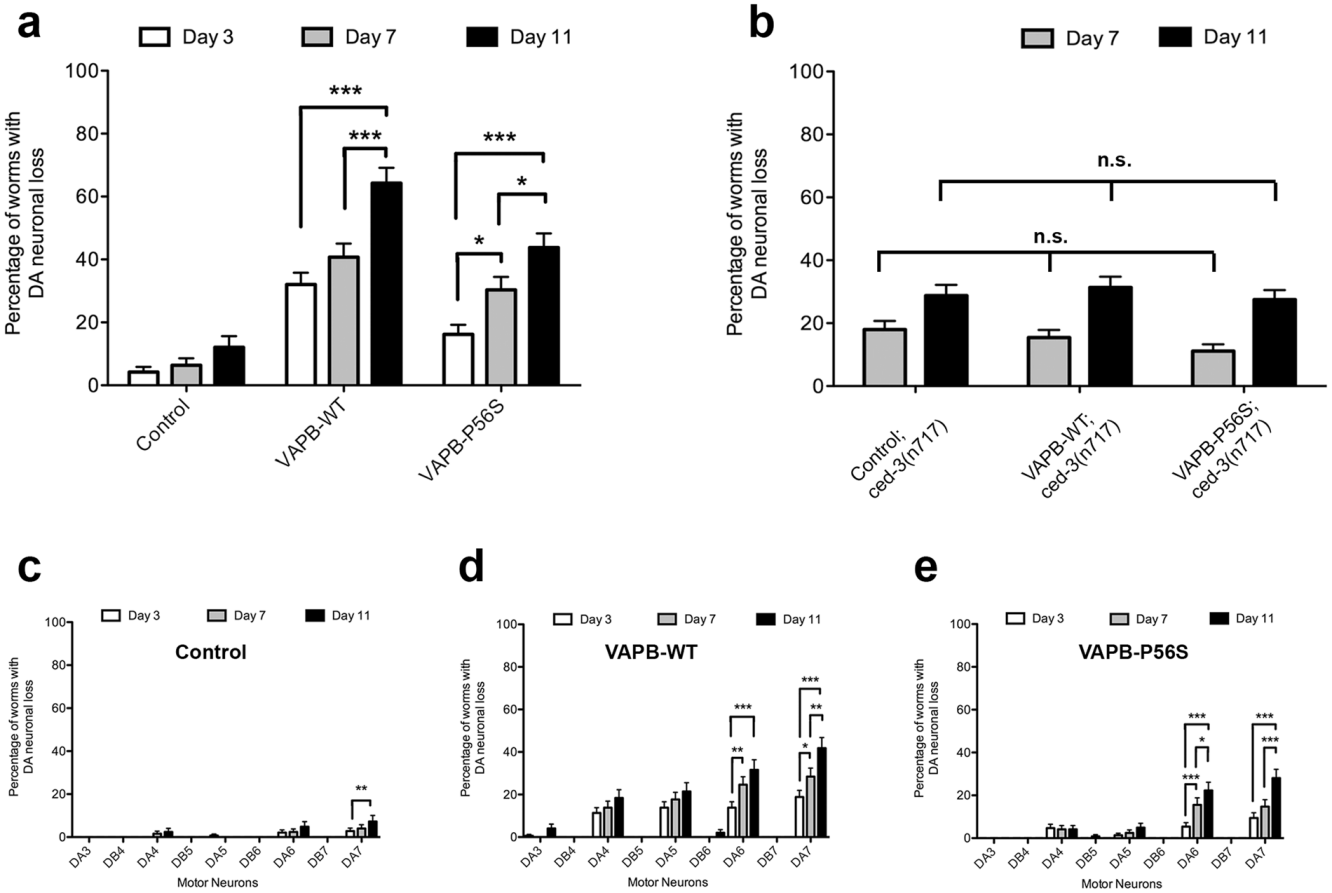
VAPB-WT and VAPB-P56S cause progressive degeneration of DA motor neurons. (**a**) Quantification of neurodegeneration in VAPB transgenic worms at Day 3, 7 and 11 of adulthood. Two-way ANOVA analysis showed that there is a significant interaction between strains and age with the incidence of neuronal loss. Further Bonferroni post-test analysis determined the significant difference between ages within the same strain. ***Represents p < 0.001, *Represents p < 0.05. Error bars represent standard error of the mean. (**b**) Frequency of neuronal loss in VAPB transgenic worms with *ced-3* mutant background. n.s. represents no significant difference between ages within each strain as determined by one-way ANOVA, followed by Bonferroni post-tests. Error bars represent standard error of the mean. Loss of individual DA and DB neurons were quantified in (**c**) control, (**d**) VAPB-WT and (**e**) VAPB-P56S worms. ***Rrepresents p < 0.001, **Represents p < 0.01, *Represents p < 0.05 as determined by one-way ANOVA, followed by Bonferroni post-tests. Average range n = 125–200, repeated 3 times. Error bars represent standard error of the mean.

Premature and progressive loss of upper and lower motor neurons is characteristic of mutant VAPB^15^. To determine whether this age-dependent loss is replicated in the worms, we quantified the loss of DA neurons at various stages of the worm’s adult life. There was no significant loss of DA neurons from Day 3 to 11 in control strains (Fig. 3a). In contrast, a significantly higher frequency of DA neuronal loss was observed in VAPB-WT (32.1% ± 3.7%) and VAPB-P56S (16.2% ± 3.0%) strains starting from Day 3. There was progressive loss of DA neurons affecting 64.3% ± 4.8% of VAPB-WT and 43.8% ± 4.5% of VAPB-P56S strains by Day 11. VAPB-WT worms showed higher incidence of DA neuronal loss compared to VAPB-P56S and control worms. This loss was restricted to DA neurons as DB neurons were largely unaffected. No obvious progressive morphological changes such as blebbing or beading were observed in the neurons and commissures. Of the DA neurons, DA6 and DA7 were the most susceptible to age-dependent loss, with an increase seen from Day 3 to 11 in both VAPB-WT and VAPB-P56S strains (Fig. 3d,e). Taken together, overexpression of human VAPB, and to a lesser extent the P56S mutant, causes axonal misguidance with DA6 and DA7 neurons the most susceptible. These DA neurons also exhibited progressive age-dependent neuronal loss, suggesting a potential relationship between axonal misguidance and neuronal death.

In our quantitative analysis, neuronal death was scored when there was a complete loss of the GFP signal in the cell body and commissures of a DA or DB motor neuron. To confirm that this truly reflects neuronal death and not simply a loss of GFP expression, we crossed the VAPB transgenic worms to a *ced-3* mutant strain, *ced-3(n717)IV*, which has a mutation in the *C*. *elegans ced-3* gene that prevents almost all programmed cell death^16^. The resulting worms with mutant *ced-3* prevented programmed cell death as indicated by significant reduction in loss of DA neurons at each life stage in the *ced-3* worms (Fig. 3b). Furthermore, the average loss observed at Day 7 and 11 was consistently lower across all VAPB strains with the *ced-3(n717)IV* background. Moreover, the incidence of neuronal loss was similar in both DA and DB neurons. Together, this indicates the loss of GFP signal we observed in the transgenic worms truly reflects neuronal loss with DA neurons inherently more vulnerable than DB neurons when human VAPB-WT or P56S mutant is overexpressed. We also examined longevity of the worms in the presence of 5′-flurodeoxyuridine (FUdR). While the VAPB-WT worms have a shorter lifespan compared to control and VAPB-P56S worms (Supplemental Fig. 2a), how this is related to overexpression of VAPB-WT remains unclear.

### Knockdown of vpr-1 in DA neurons causes age-dependent locomotor defects

Our transgenic VAPB worms produced an unexpected axonal misguidance phenotype, which is a developmental defect not seen in human ALS8. This prompted us to generate an alternative worm model targeting VAPB function in motor neurons. Previous studies have shown decreased levels of VAPB in spinal cords of ALS patients^17^ as well as in motor neurons derived from iPSCs of ALS8 patients^18^. Furthermore, the aggregation-prone VAPB-P56S is thought to act in a dominant negative fashion by recruiting endogenous VAPB-WT to the protein aggregates^19^. Together, this indicates that expression of mutant VAPB generates a loss of function phenotype. To recapitulate this pathogenic mechanism, we generated a second *C*. *elegans* model by knocking down *vpr*-*1*, the *C*. *elegans* ortholog of VAP. Because knocking out *vpr*-*1* is embryonic lethal, and *C*. *elegans* neurons are resistant to RNA interference (RNAi) knockdown via feeding^20^, we generated a strain with inducible knockdown of *vpr-1* only in DA motor neurons. We reconstituted RDE-1, an Argonaute protein essential for RNAi, activity in DA neurons of an *rde-1* null background strain by placing it under the control of an *unc-4* promoter. Next, plasmids with the heat-inducible *hsp-16* promoter driving *vpr-1* sense and anti-sense transcripts were introduced^21^. This causes heat-inducible knockdown of *vpr-1* only in DA motor neurons due to its reconstituted RDE-1 activity while all other cells remain unaffected. As control, the *unc-4p::rde-1* plasmid was replaced with the empty vector, and thus control worms remained resistant to RNAi knockdown even with heat shock.

**Figure 4:**
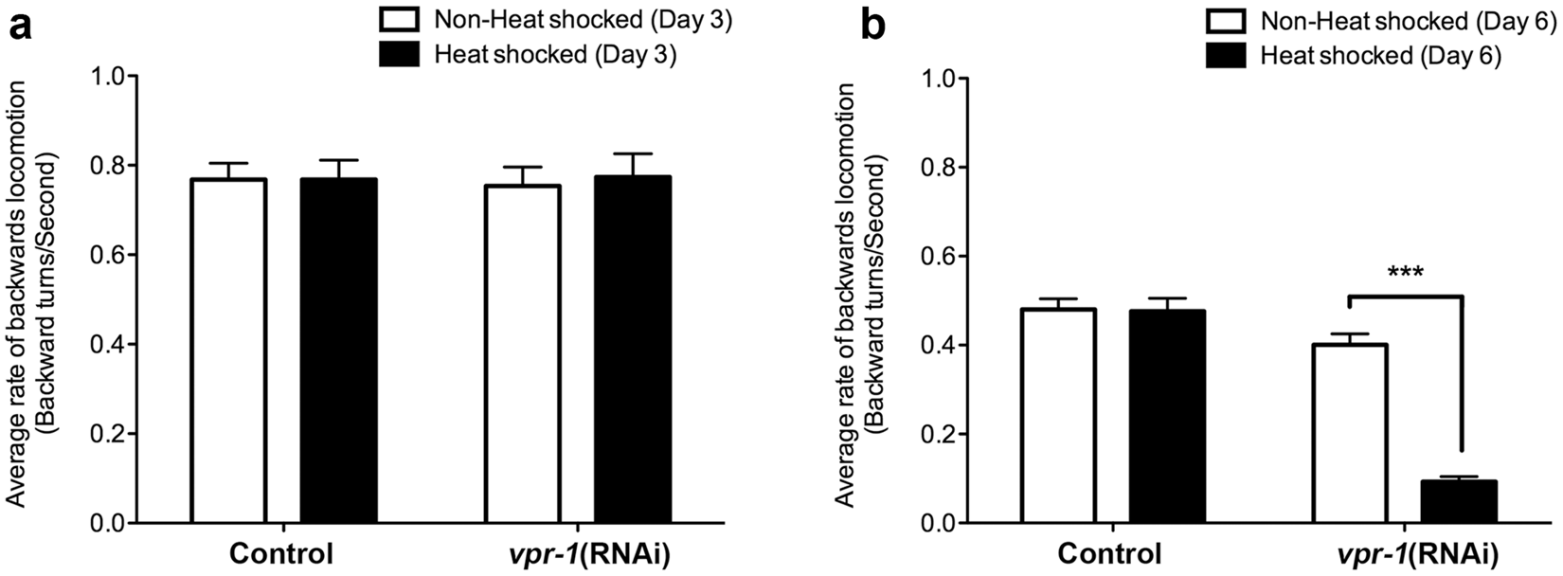
v*pr-1* knockdown in DA neurons results in a slowed rate of backward locomotion. Average rate of backward locomotion of (**a**) Day 3 and (**b**) Day 6 *vpr-1*(RNAi) knockdown strains following stimulus. ***Represents p < 0.001 as determined by one-way ANOVA, followed by Bonferroni post-tests. Average range n = 80–120, repeated 3 times. Error bars represent standard error of the mean.

We first assessed the presence of backward locomotion defect through a reversal behaviour assay. The worms were heat shocked for 2.5 hours at alternating days starting from larval L4 stage. This bypasses embryonic lethality and developmental defect associated with loss of *vpr-1*. During early adulthood (Day 3), the rates of backward locomotion of both the control (0.77 ± 0.04 turns/second) and *vpr-1*(RNAi) (0.75 ± 0.04 turns/second) were not significantly different, regardless of heat inducible *vpr-1* knockdown (Fig. 4a). As the worms age, backward locomotion rates slowed considerably at Day 6 with rates for the control and *vpr-1*(RNAi) worms at 0.48 ± 0.02 turns/second and 0.40 ± 0.02 turns/second, respectively (Fig. 4a vs. 4b). The rate of backward locomotion remained unchanged in control worms regardless of whether the strain was heat shocked (0.48 ± 0.03 turns/sec) or not (0.48 ± 0.02 turns/sec; p > 0.05), indicating the heat shock protocol had no adverse effect on the locomotor behaviour. In contrast, the rate of backward locomotion of *vpr-1*(RNAi) worms subjected to heat-inducible knockdown was over four times slower than worms not subjected to heat-induced *vpr-1* knockdown (0.09 ± 0.01 turns/sec compared to 0.40 ± 0.02 turns/sec, respectively, p < 0.001). Thus, heat inducible knockdown of *vpr-1* in adult DA neurons gave rise to a significant decrease in backward locomotion.

**Figure 5:**
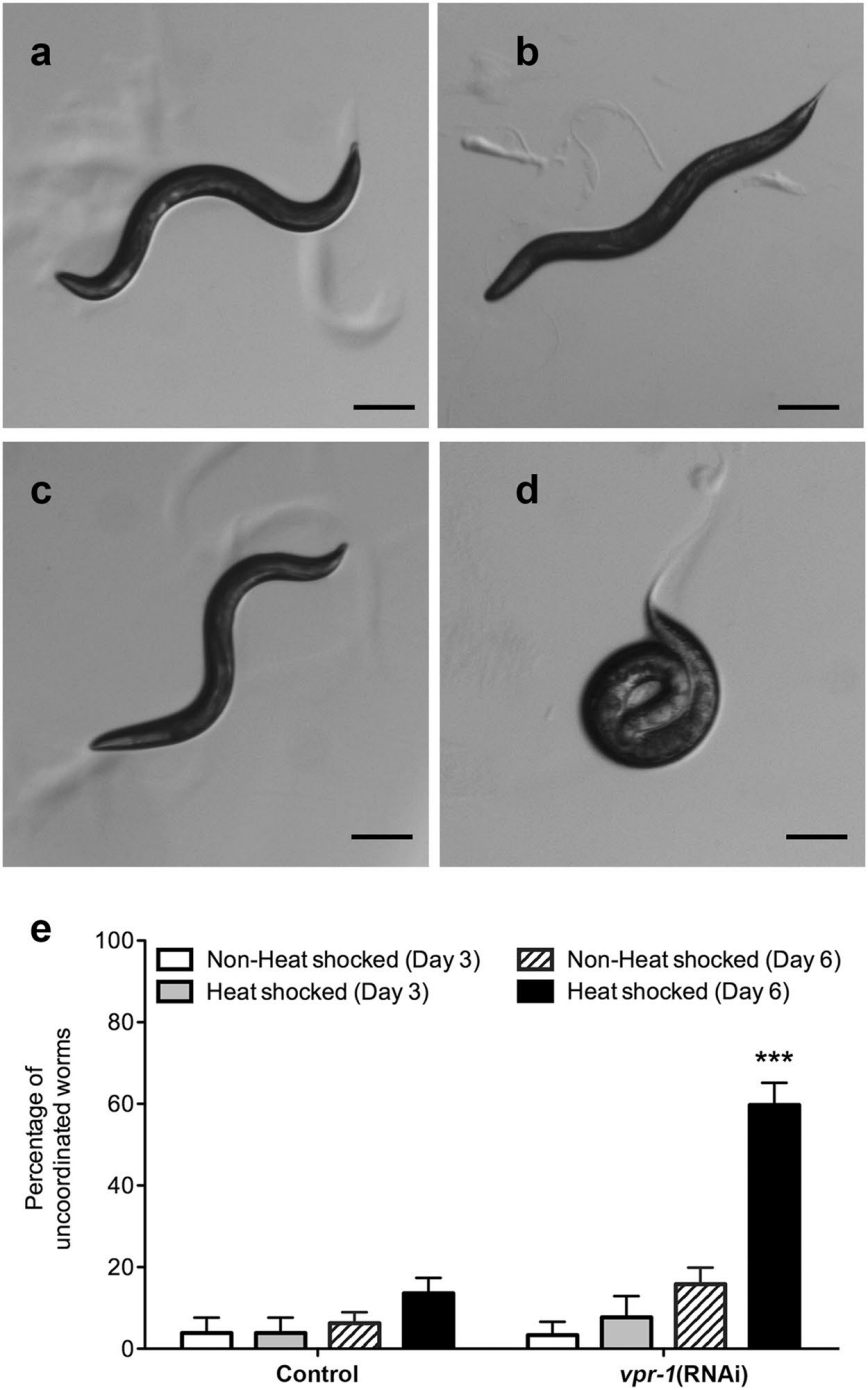
*vpr-1* knockdown in DA neurons leads to uncoordinated phenotype. Representative images of adult Day 6 (**a**) non-heat shocked control, (**b**) heat shocked control, (**c**) non-heat shocked *vpr-1*(RNAi), and (**d**) heat shocked *vpr-1*(RNAi) worms after a stimulus was applied to the head to trigger backward locomotion. Scale bar = 100 μm. (**e**) Incidences of uncoordination characterized by a coiling phenotype by Day 6. Two-way ANOVA was used to determine the significant interaction between strain and treatment with the incidence of uncoordination. Further Bonferroni post-test analysis was conducted to show difference between the treatments within each strain. ***Represents p < 0.001. Average range n = 80–120, repeated 3 times. Error bars represent standard error of the mean.

In addition to the slower rate of backward locomotion following heat shock induced *vpr-1* knockdown in DA neurons, an abnormal uncoordinated phenotype was observed during the reversal behaviour assays. *C*. *elegans* typically move in an undulating, sinusoidal backward fashion away from the stimulus^22^. However, some *vpr-1*(RNAi) worms exhibited a characteristic uncoordinated phenotype such as coiling or partial body movements (Fig. 5d). At Day 3, the frequency of *vpr-1*(RNAi) worms with this uncoordinated phenotype was low and not significantly different from control worms regardless of heat shock treatment. However, by Day 6, 59.8% ± 5.4% of heat shocked *vpr-1*(RNAi) worms showed uncoordinated coiling phenotype (Fig. 5e), which was significantly higher than non-heat shocked *vpr-1*(RNAi) worms (15.9% ± 4.0%, p < 0.001). This indicates that the slowed rate of backward locomotion and uncoordinated phenotype are dependent on knockdown of *vpr-1* and progressively worsen with age.

### Knockdown of vpr-1 in DA neurons causes progressive degeneration of DA motor neurons

**Figure 6:**
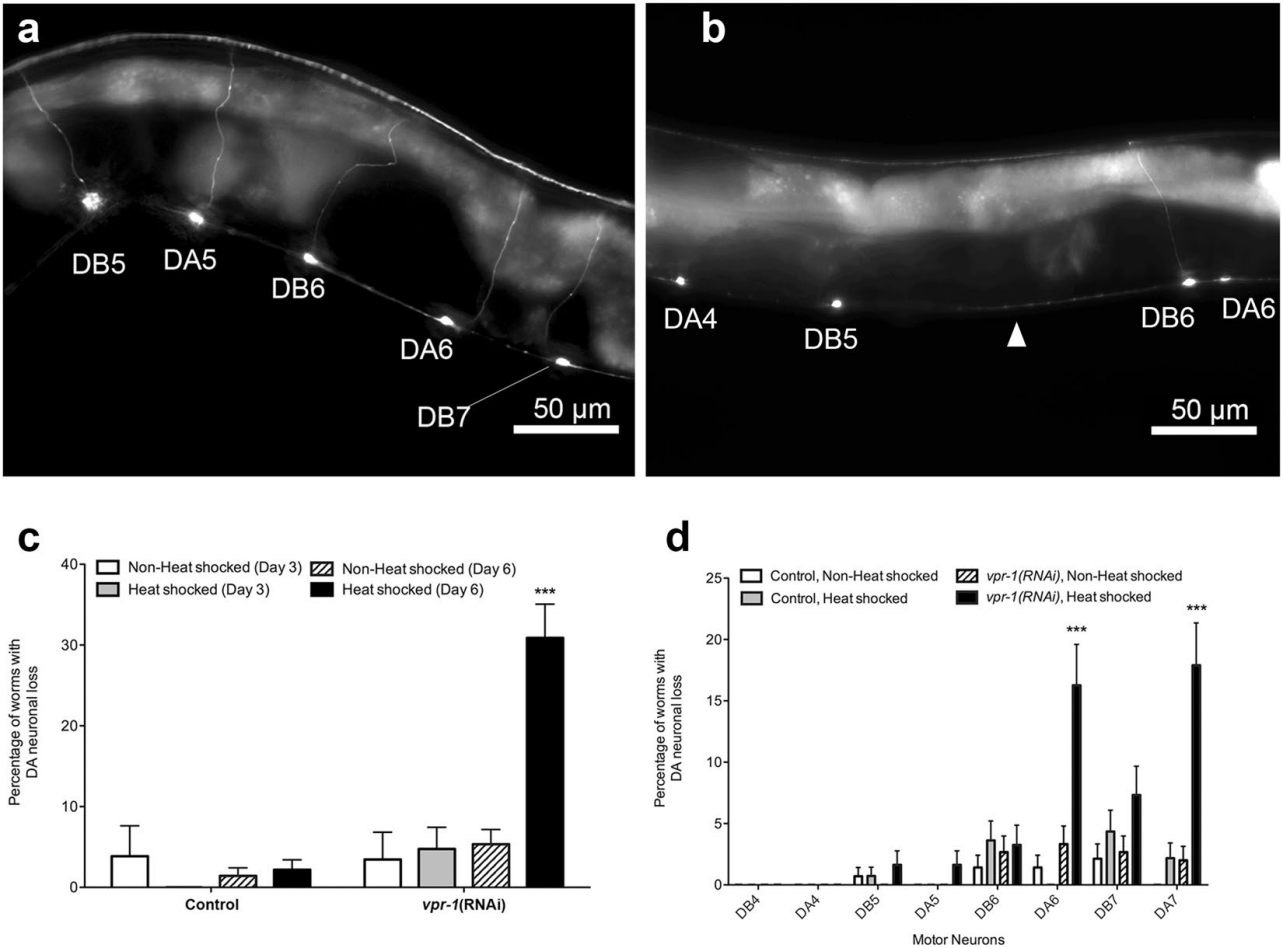
v*pr-1* knockdown in DA neurons induces age-dependent neuronal loss by Day 6 of adulthood. Representative images of DA and DB neurons of heat shocked (**a**) control and (**b**) *vpr-1*(RNAi) at Day 6 are shown. Arrowheads represent missing DA neurons. Scale bar = 50 μm. (**c**) Frequency of worms with neuronal loss at Day 3 and Day 6 with or without heat shock treatment. Rde-1 activity was restored in DA neurons of *vpr-1*(RNAi) worms but not in control worms. (**d**) Incidence of individual DA and DB motor neuronal loss in control and *vpr-1*(RNAi) knockdown worms at Day 6 with or without heat shock treatment. Two-way ANOVA was used to determine the significant interaction between strain and treatment with the incidence of neuronal loss. Further Bonferroni post-test analysis was conducted to show difference between the treatments within each strain. ***Represents p < 0.001. Average range n = 100–150, repeated 3 times. Error bars represent standard error of the mean.

We next examined whether the locomotion defect correlates with motor neuron loss. The knockdown strains were crossed to *unc-129p::GFP* reporter to visualize the DA and DB neurons (Fig. 6a and b). Neither control nor *vpr-1*(RNAi) strains showed significant differences in the incidence of DA neuronal loss at Day 3. However, by Day 6, the frequency of worms with neuronal loss was significantly higher in *vpr-1*(RNAi) worms following heat-induced *vpr-1* knockdown (30.9% ± 4.2%) when compared to the same strain without the heat shock induction of *vpr-1* knockdown (5.3% ± 1.8%, p < 0.001). This DA neuronal loss was not observed in control worms, which exhibited only low frequencies of DA neuronal loss at Day 6 with or without heat induction (2.2% ± 1.2% and 1.4% ± 1.0%, respectively) (Fig. 6c). Thus, the loss of DA neurons is not only dependent on the knockdown of *vpr-1* but is also age-dependent, and correlated with the onset of backward locomotion defect. There was no significant difference in lifespan between control and *vpr-1*(RNAi) strains either kept continuously at 20 °C or subjected to heat shock every 2 days (Supplemental Fig. 1b).

The posterior DA neurons, DA6 and DA7, appear to be the most susceptible to age-dependent neuronal loss following heat shock-induced *vpr-1* knockdown in the *vpr-1*(RNAi) strains (16.3% ± 3.33% for DA6 and 17.39% ± 3.5% for DA7). Consistently across control or *vpr-1*(RNAi) worms with or without the heat shock induction of *vpr-1* knockdown, the frequency of worms exhibiting DA6 or DA7 loss at Day 6 was significantly higher than other DA neurons (Fig. 6d). Thus, the enhanced susceptibility of the posterior DA neurons, DA6 and DA7, is similar to that observed in the VAPB transgenic worms.

### PIK-93 treatment delays DA neuronal loss in C. elegans with vpr-1 knocked down

Previous studies have shown that depletion of VAPs by RNA interference increases the levels of PtdIns4P, diacylglycerol, and sphingomyelin in the Golgi membranes, and leads to substantial inhibition of Golgi-mediated transport events^23, 24^. Moreover, inhibiting the synthesis of PtdIns4P reduced the severity of the VAPB-P56S-induced membrane expansions and partially restored Emerin trafficking to the nuclear envelope^25^. Thus, high levels of PtdIns4P are associated with the loss of VAPB phenotype. We decided to test if reducing PtdIns4P levels might rescue DA neuronal loss in the *vpr-1* knockdown worms. We tested the effects of the PtdIns4-kinase III β inhibitor PIK-93 on the frequency of DA neuronal loss and backward locomotion of the *vpr-1*(RNAi) worms. To determine the optimal dosage of PIK-93, varying concentrations of the inhibitor were given on Day 5, one day before the onset of DA neuronal loss and scored on Day 6. PIK-93 at 250 nM was the most effective in reducing the frequency of DA neuronal loss (Supplemental Fig. 3). Subsequently, worms were treated with 250 nM of PIK-93 or DMSO control, and DA neuronal loss was scored on Day 6, 8 and 10. The drug was replenished daily for the duration.

**Figure 7:**
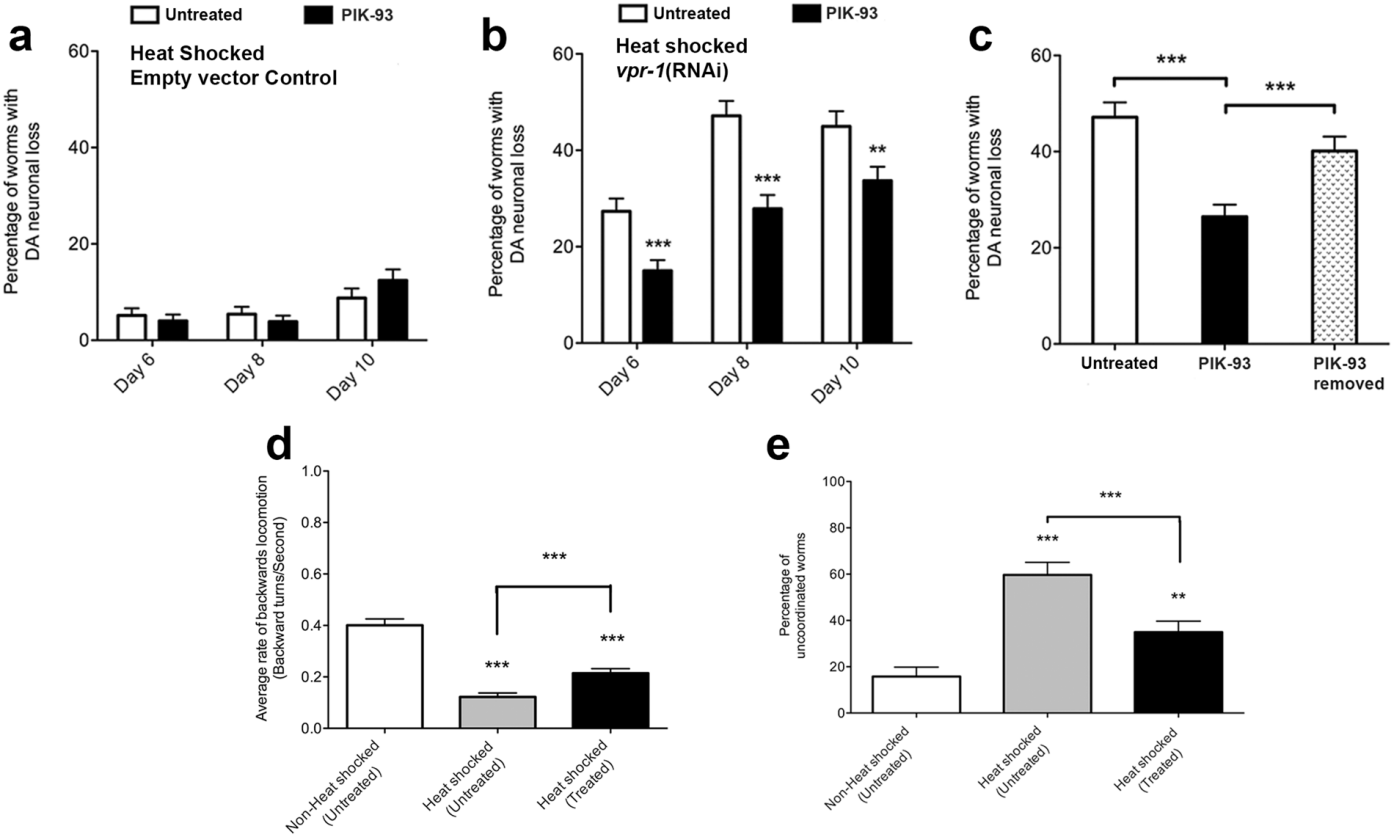
PIK-93 treatment delays DA neuronal loss and partially restores backward locomotion defects in *C. elegans* with *vpr-1* knocked down. Effect of sustained 250 nM PIK-93 treatment on DA neuronal loss in (**a**) heat shocked control worms and (**b**) heat shocked *vpr-1*(RNAi) worms from Day 6 to 10. ***Represents p < 0.001, **Represents p < 0.01 as determined by Student’s *t*-test. Error bars represent standard error of the mean. (**c**) Frequency of neuronal loss in heat shocked *vpr-1*(RNAi) by Day 8 when PIK-93 was removed for 24 hours following an initial 24-hour treatment at Day 5. ***Represents p < 0.001 as determined by Student’s *t*-test. Average range n = 200–250, repeated 3 times. Error bars represent standard error of the mean. (**d**) Rate of backwards locomotion and (**e**) frequency of uncoordinated *vpr-1*(RNAi) worms at Day 6 following no treatment or sustained PIK-93 treatment. ***Represents p < 0.001 as determined by Student’s *t*-test. Average range n = 100–150, repeated 3 times. Error bars represent standard error of the mean.

PIK-93 had no effect on DA neuronal loss in heat shocked control worms when compared to DMSO control (Fig. 7a), indicating that the drug has no adverse effect on the motor neurons. When given to *vpr-1* knockdown worms, PIK-93 significantly reduced the frequency of worms with DA neuronal loss from Day 6 to 10 (Fig. 7b). Worms treated with PIK-93 exhibited significantly lower instances of DA neuronal loss at Day 6 and Day 10 (15.0% ± 2.2% and 33.7% ± 2.9%, respectively, p < 0.001) when compared to mock treated control (27.3% ± 2.7% and 44.9% ± 3.2% at Day 6 and Day 10, respectively, p < 0.001). The inhibitor had no effect on DB neuron survival. Together, this indicates that inhibiting PtdIns 4-kinase activity reduced the frequency of DA neuronal loss caused by the knockdown of *vpr-1* in these neurons.

Specificity of this PIK-93 effect was further tested by removal of the drug for 24 h at Day 7. While *vpr-1*(RNAi) worms receiving uninterrupted PIK-93 treatment showed reduced frequency of DA neuronal loss compared to mock treated control (Fig. 7c), removal of PIK-93 for 24 h resulted in significantly higher frequency of DA neuronal loss. Thus, the reduction in DA neuronal loss observed in *vpr-1* knockdown worms was dependent on PIK-93. It suggests the inhibitor must be continuously present to sustain DA neuron survival.

Since significant neuronal loss began by Day 6, reversal behaviour assays were conducted at Day 6 following PIK-93 treatment. Without PIK-93 treatment, the rate of backward locomotion of heat-induced *vpr-1*(RNAi) worms was over four times slower than worms not subjected to heat-induced *vpr-1* knockdown (0.09 ± 0.01 turns/sec compared to 0.40 ± 0.02 turns/sec, respectively, p < 0.001). However, the rate of backward locomotion of these worms was significantly higher when treated with PIK-93 (0.21 ± 0.02 turns/sec, p < 0.001) compared to untreated worms. While PIK-93 treatment did not completely restore backward locomotion to control levels, this single 24 h treatment resulted in a two-fold improvement over the untreated control (p < 0.001). We also monitored the effect of PIK-93 on the uncoordinated phenotype, which was observed in 59.8% ± 5.4% of heat shocked *vpr-1*(RNAi) worms on Day 6. Treatment with PIK-93 for 24 h significantly reduced this to 35.0% ± 4.8% (p < 0.01). Therefore, treatment with PIK-93 partially rescued the backward locomotion defect and uncoordinated phenotype of the *vpr-1*(RNAi) worms.

## Discussion

Since ALS is characterized by progressive degeneration of motor neurons, we generated two *C*. *elegans* models of VAPB to determine its role in motor neuron survival. The *unc-4* promoter, which is expressed in DA but not DB neurons, was used to drive differential expression of the transgenes in both models. DA neurons, which control backward locomotion, were specifically targeted because forward-directed locomotion is essential for survival and their loss could affect the worm’s ability to feed^26^. This strategy also allows the use of DB neurons as internal controls for neuronal survival. In the first model, human VAPB-WT or mutant P56S were expressed in DA motor neurons. Reversal behaviour assays revealed the VAPB-WT and VAPB-P56S worms had significantly slower rates of backward locomotion compared to control (Fig. 1). No significant difference in backward-directed locomotion was observed between at Day 3 in the VAPB-WT and VAPB-P56S worms, which indicate that the P56S mutation retains some VAPB function, as suggested by others^4, 27^. This suggests that cellular expression of VAPB is maintained at an optimal level and overexpression of the protein is not a desirable trait.

The morphology of DA neurons was also affected by overexpression of human VAPB-WT or mutant P56S. In control worms, both DA and DB neurons exhibited the distinctive parallel axons projecting from ventral to dorsal midline. However, DA axons from VAPB-WT and, to lesser extent, VAPB-P56S did not reach the dorsal side. Instead, the DA commissures were mistargeted or misguided. DB neurons, which do not express the transgenes, were unaffected (Fig. 2). In addition to DA axonal misguidance, there was an increased frequency of DA neuronal loss from adult Day 3 to 11 in both VAPB-WT and VAPB-P56S. DA neuronal loss increased with age in both transgenic strains with the VAPB-WT expressing strain consistently exhibiting the highest frequency of worms with neuronal loss (Fig. 2d). Interestingly, there is no reported axonal misguidance in human ALS8 cases, and only minor axonal defects in zebrafish^28^. Thus, it is possible that the DA axonal misguidance in the VAPB transgenic worms may be limited to nematodes. This is likely a developmental defect as DA neurons, one of only three motor neuron classes that are present along the ventral nerve cord upon hatching^29^, are born just after the 300-minute mark of embryogenesis and extend commissures to the dorsal cord during mid-embryogenesis. Axons are directed by extracellular guidance cues, such as UNC-6/netrin, Slt-1/slit, VAB-1/Eph Receptor (EphR) to reach and functionally connect with their targets. Previous studies have shown that VAB-1 EphR functions to prevent abnormal axon crossing at the ventral midline^30, 31^ and mutations in EphR cause defects in axonal guidance during development^32^. The MSP domain of VAP appears to bind EphR such that loss of *vpr-1*, the VAP orthologue in *C*. *elegans*, results in in distal-tip cell (DTC) migration defect^8^. Thus, *unc-4p* driven overexpression of VAPB-WT or VAPB-P56S when processed could potentially interfere with signaling and lead to axonal mistargeting of DA neurons during this developmental stage. The P56S mutation in the MSP domain of VAPB is thought to disrupt processing of the MSP domain or its secretion, which could potentially account for the reduced severity of the guidance defect. Alternatively, overexpression of human VAPB-WT or P56S may interfere with the secretion of morphogens and/or surface presentation of their receptors at critical stages of neuronal development.

Since the loss of GFP signal in the commissure and cell body was used as an indicator of DA neuronal loss, crossing the transgenic worms into a *ced-3* mutant background validated apoptotic neuronal loss. *ced-3* encodes a caspase required for apoptosis and mutation of *ced-3* prevents almost all programmed cell death^16^. Given that neuronal loss was blocked in transgenic worms with the *ced-3* background, the observed loss of GFP signal in the DA neurons truly reflects neuronal death (Fig. 3). Together, it is likely that axonal misguidance is a contributing factor in the backward locomotion defect in the transgenic worms. Misguidance could also affect innervation and neuronal function that then lead to enhanced degeneration.

Because of the unexpected axonal guidance defect in the transgenic worms, a knockdown model of the VAP orthologue, *vpr*‐*1*, was subsequently generated to recapitulate the loss of VAPB function characteristic of ALS8. Previous studies have shown that the aggregation-prone VAPB-P56S acts in a dominant negative fashion by dimerizing with endogenous VAPB-WT, causing a loss of VAPB function that then leads to defects in the ER and nuclear envelope^4, 5, 19, 33^. Our heat shock-inducible knockdown model effectively bypassed the developmental defect in the transgenic model. It recapitulated age-dependent onset of motor defect seen in human. No significant locomotor defect was observed in young worms at Day 3 following heat shock induced *vpr-1* knockdown in the DA neurons, but there was a significant increase in the frequency of worms exhibiting uncoordinated backward-directed locomotion by Day 6 (Fig. 4). The non-heat shocked *vpr-1*(RNAi) worms also showed no detectable backward locomotion defect, suggesting that leakiness of the *hsp-16*.2 promoter is minimal. In addition, uncoordination is a common behavioural phenotype where the *vpr-1*(RNAi) worm makes a few backward body bends but comes to rest in a coiled posture, and remains stationary in this position for a period of time (Fig. 5). This defect may be the result of a disruption of neuronal input to the body wall and interruption of the typical pattern of alternating waves of contraction along the body wall^34^. Thus, this phenotype represents a more severe disruption of coordinated neuronal activity. The increased DA neuronal loss in the *vpr-1*(RNAi) worms (Fig. 6) likely leads to loss of innervation of segments of the dorsal muscle thereby affecting the ability of the worm to make alternating waves of contraction along the body wall for its typical sinusoidal motion.

In terms of neuronal specificity, DA6 and DA7 were the most vulnerable DA neurons in both the human VAPB transgenic model and the *vpr-1* knockdown model (Figs 3 and 6). These neurons are also most susceptible to age-dependent neuronal loss, although the reason remains unclear. It is possible that increased vulnerability in these neurons is due to higher expression of the transgenes or greater knockdown. These subtle changes in expression may underlie variable vulnerability in the motor neuron population. It is clear that expression from the *unc-4* promoter is confined to the DA neurons as there was little to no defect observed in the DB neurons. This was reflected by the lack of any detectable forward locomotor defect in the two models.

Overexpression of VAPB-P56S or knockdown of VAPB results in the accumulation of large vacuole-like membrane expansions of the endoplasmic reticulum Golgi intermediate compartment (ERGIC)^5^. This not only sequesters numerous ER but also nuclear envelope proteins. One of the proteins affected is SAC1, a lipid phosphatase that hydrolyzes PtdIns4P^35^, which then leads to an increase in the levels of PtdIns4P in Golgi membranes^36^. Overexpressing SAC1 is ineffective in reducing PtdIns4P in VAPB knockdown cells as the phosphatase is also trapped in the expanded membrane structures. An alternative strategy is to reduce the PtdIns4P levels by inhibiting its synthesis. This was shown to partially rescue the trafficking defect caused by knockdown of VAPB^25^. When the PtdIns 4-kinase III β inhibitor, PIK-93, was given prior to the onset of neuronal loss at Day 5, it significantly reduced the frequency of DA neuronal loss in *vpr-1*(RNAi) worms from Day 6 to 10 (Fig. 7b). Uninterrupted treatment with PIK-93 is essential as withdrawal of the drug for 24 h resulted in resumption of DA neuronal loss (Fig. 7c). Since PIK-93 treatment also led to improvement of the backward locomotion defect and uncoordinated phenotype in the *vpr-1*(RNAi) worms, it suggests that the surviving motor neurons remain functional to some extent. This indicates that reducing the intracellular levels of PtdIns4P can bypass the loss of *vpr-1* to maintain DA motor neuron function and viability at this adult stage. It raises the possibility that this model can be used to test therapeutic strategies targeting PtdIns4P to delay neuronal loss and maintain motor neuron functions.

## Materials and Methods

### Strains

*C*. *elegans* strains were maintained at 20 °C using standard methods^37^. Worm strains used in this study were obtained from the *C*. *elegans* Genetics Center (University of Minnesota, Minneapolis), including: N2, *rde-1(ne219) V*, *ced-3(n717) IV*.

Transgenic VAPB strains (VAPB-WT, VAPB-P56S, control) were generated through microinjection of DNA solutions containing the specific construct (*unc-4p::VAPB-WT*, *unc-4p::VAPB-P56S*, or empty *unc-4p* vector) along with a phenotypic marker, *odr-1p::DsRed*, into N2 worms using standard methods^38, 39^. The resulting worms were crossed with *unc-129p::GFP* reporter strain^40^ to visualize the DA and DB motor neurons. The *vpr-1*(RNAi) knockdown strains were also generated through microinjection of *unc-4p::rde-1*, *hsp-16*.*2p::vpr-1 s*, *hsp-16*.*2p::vpr-1 as* along with the marker *myo-2p::dsRed* into *rde-1(ne219); unc-129p::GFP*. *vpr-1*(RNAi). Multiple lines were generated and strains behaving similarly were kept for further analysis. For longevity analysis, 120 µM of 5′-flurodeoxyuridine (FUdR) was added to the media. The worms were scored every 2 days, and considered dead if the pharynx does not pump and they do not respond to prodding with a pick.

### Molecular cloning

The cDNAs containing FLAG-tagged human VAPB-WT and VAPB-P56S were PCR amplified from FLAG-tagged human VAPB-WT and FLAG-tagged human VAPB-P56S constructs^33^. These were subcloned into the *unc-4p*, and the clones were confirmed by DNA sequencing. Plasmid pPD49_78 with *hsp-16*.*2p* (Dr. Andrew Fire, Addgene plasmid 1447) expressing sense and antisense *vpr-1* was used to induce RNAi knockdown of *vpr*‐*1* at specific stages using heat shocks. *unc-4p::rde-1* was used to restore RDE-1 function and limit *vpr-1* RNAi knockdown to DA neurons specifically. The empty *unc-4p* plasmid was used as control so that RDE-1 function is not restored in DA neurons.

### RNAi treatment

Heat shocks were performed on *vpr-1* knockdown model worms, *vpr-1*(RNAi), to induce knock down of *vpr-1* in DA neurons. Adult *vpr-1*(RNAi) or control worms were bleached to harvest the eggs, and the larvae were heat shocked 24 h later at 35 °C for 2.5 hours. The heat shock was repeated at stages L4, adult Day 2, adult Day 3, and adult Day 5. For longer studies, heat shocks were continued every 48 h until they were scored.

### Backward locomotion assay

An immediate reversal from forward to backward locomotion was induced by gently prodding the worm with the thin platinum wire of a worm picker just behind the pharynx (Adapted from WormBook^41^). One full body bend was defined as when the part of the worm just behind the pharynx reached a maximum bend in the opposite direction from the bend that was last counted. Worms that coiled up or only made partial body movements were deemed “uncoordinated”. The reversal behavior of adult VAPB transgenic worms of each condition (control, VAPB-WT and VAPB-P56S) and *vpr-1*(RNAi) worms was scored on unseeded NGM plates at 20 °C under a Leica MZ6 stereomicroscope. Each trial was repeated three times for each worm in all locomotor behavior experiments.

### PIK-93 treatment

Worms grown on NGM were transferred to liquid culture containing OP50 and 250 nM of PtdIns 4-kinase III β inhibitor, PIK-93, at Day 5 of adulthood. Worms were scored at 20 °C for neuronal loss after 24 h of PIK-93 drug treatment and for backward locomotion. For longer studies, the worms were supplemented with PIK-93 every 24 h.

### Microscopy

Zeiss Stereo Discovery.V20 Microscope was used to capture brightfield images of uncoordinated worms using 1x objective at magnifications of 7.5x to 100x. Fluorescence microscopy on Zeiss AxioImager.M2 epifluorescence microscope was used to visualize individual neurons. Since DA and DB neurons express GFP under the *unc-129* promoter, live neurons were scored for a visible GFP signal. A complete absence of GFP signal indicated a loss of neurons. Neuronal loss was further confirmed through crossing the VAPB transgenic worms to a *ced-3* mutant strain, *ced-3(n717)IV*. Images were taken with 10 x and 20 x objectives using the Zeiss AxioVision software version 4.8. Stacks of DA and DB neuron images with an interval of 0.5 μm between each slice were collapsed into a single image and processed with Image J (NIH, Bethesda, MD, USA).

For axonal misguidance and neuronal loss, transgenic VAPB and *vpr*-*1*(RNAi) worms were scored at day 3, 6, 7, or 11 of adulthood. The worms were immobilized in M9 with 30 mM levamisole and mounted on slides with 2% agarose pads. For each strain, 100–200 worms were scored and repeated 3 times. The results are shown as mean ± standard error.

### Statistical analysis

All statistical analyses were conducted using GraphPad Prism 5.0a. Results were deemed statistically significant when p < 0.05.

## Electronic supplementary material

Supplemental Information
