# Supplementary material for: Mitigating Motor Neuronal Loss in C. elegans Model of ALS8
Source: Sci Rep. 2017 Sep 14;7:11582. doi: 10.1038/s41598-017-11798-6 (PMC5599522; doi:10.1038/s41598-017-11798-6)
Supplement: Supplementary file 1 — Supplemental Information [file 41598_2017_11798_MOESM1_ESM.pdf]

# Mitigating Motor Neuronal Loss in *C. elegans* Model of ALS8

Wendy Zhang, Antonio Colavita and Johnny K. Ngsee*
Neuroscience, Ottawa Hospital Research Institute, Cellular and Molecular Medicine, University of Ottawa, 451 Smyth Road, Ottawa, Ontario, Canada, K1H 8M5

Corresponding author: Johnny K. Ngsee, jngsee@uottawa.ca

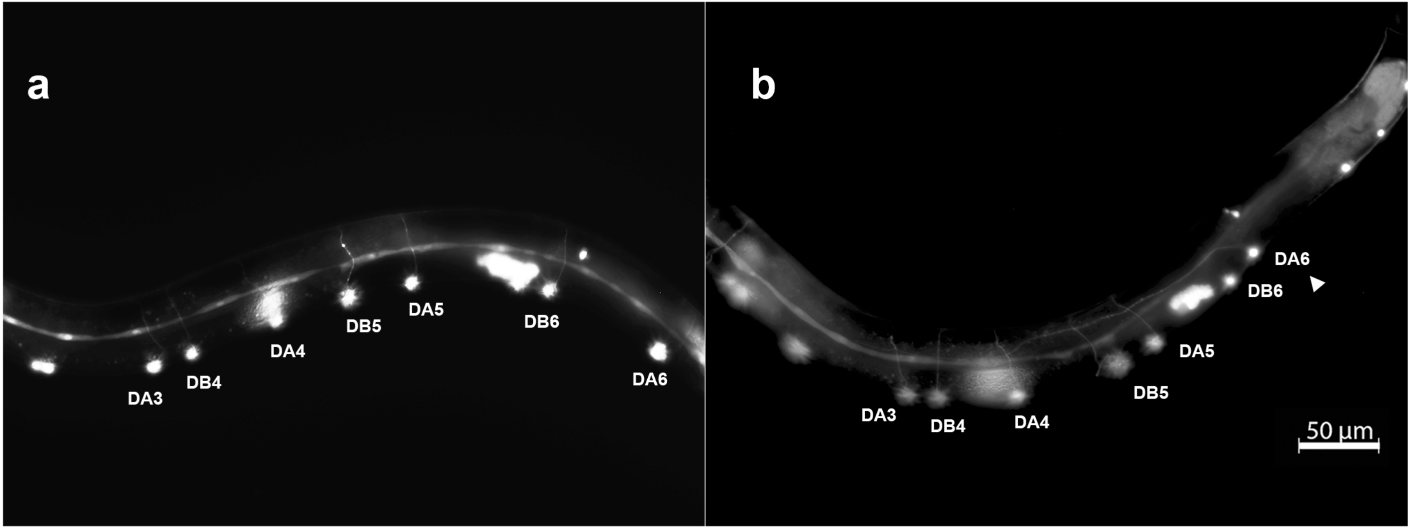


**Supplemental Figure 1. Axonal misguidance in DA motor neurons of VAPB-WT worms at larval L4.** Representative images of DA and DB neurons of (a) VAPB-WT worm with normal axons and (b) VAPB-WT worm exhibiting misguided DA6 axon. Arrowhead represents misguided DA6 axon. Note the axon failed to reach dorsal midline and projected anteriorly along the lateral body wall.

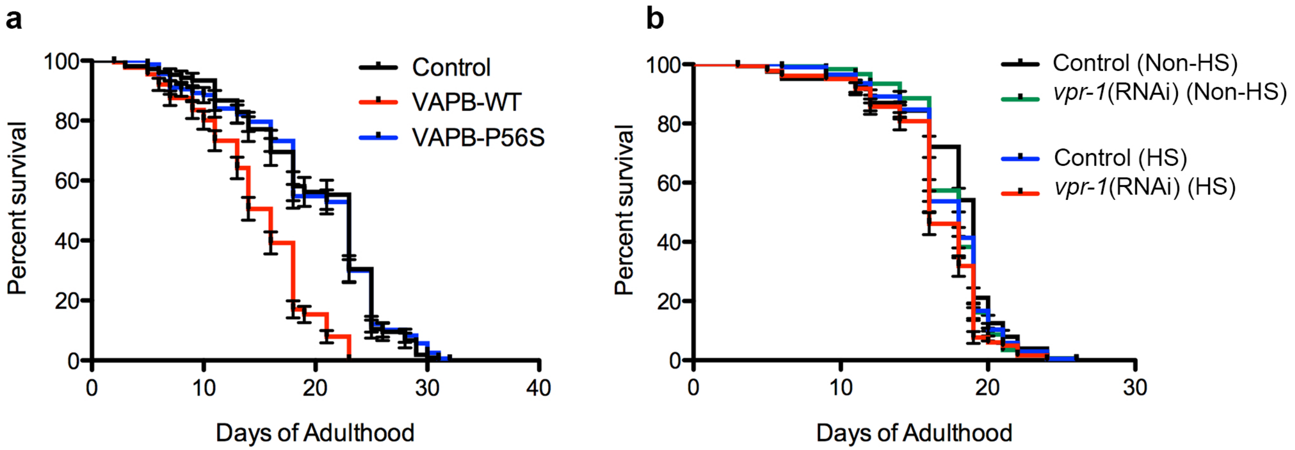


**Supplemental Figure 2. Lifespan analysis.** (a) The VAPB transgenic worms were maintained in 5'-flurodeoxyuridine (FUdR) media and scored for live/dead worms every 2 days. (b) Control and vpr-1(RNAi) worms were maintained in FUdR media and scored for live/dead worms every 2 days. The worms were kept continuously at 20°C (Non-heat shocked, non-HS) or heat shocked (HS) for 2.5 h every 2 days. Survival curves were produced and compared using the Log-rank (Mantel-Cox) test ($p<0.001$ for VAPB-WT compared to control). Average range n=100-150, repeated 3 times. Error bars represent standard error of the mean.

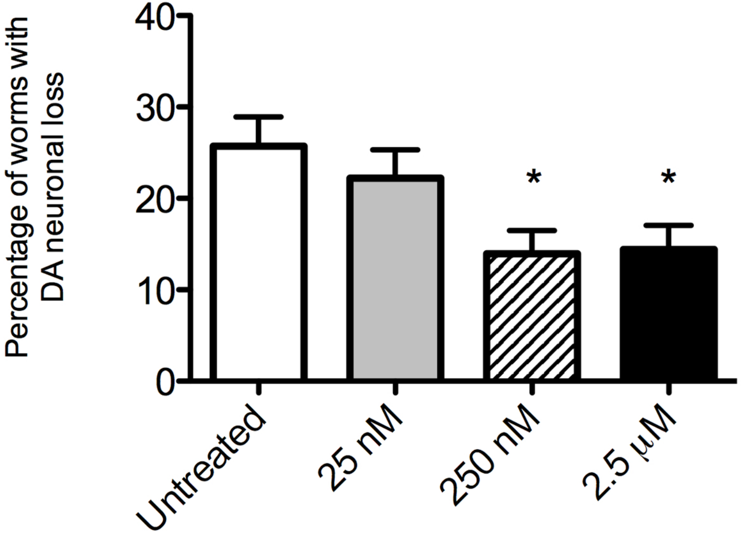


**Supplemental Figure 3. Effect of PIK-93 dosage on DA motor neuron loss.** Heat shocked vpr-1(RNAi) worms were treated with indicated doses of PIK-93 at Day 5 and DA motor neuron loss was scored at Day 6. Untreated worms were given vehicle DMSO. * represents $p<0.05$ compared to untreated as determined by Student's t-test. Average range n=100-150, repeated 3 times. Error bars represent standard error of the mean.
